# Preparation of an epitope-based recombinant diagnostic antigen specific to anti-phospholipase A_2_ receptor 1 antibodies

**DOI:** 10.1186/s12896-018-0448-8

**Published:** 2018-05-29

**Authors:** Hua Li, Yonghua Xu, Weiming Yang, Xiaohong Huang

**Affiliations:** 1 Jiangxi Province Key Laboratory for Laboratory Medicine, Department of Clinical Laboratory, The Second Affiliated Hospital of Nanchang University, Nanchang, 330006 China; 2 Jiangxi Maternal and Child Health Hospital, Nanchang, China

**Keywords:** Phospholipase A_2_ receptor 1 (PLA_2_R1), Prokaryotic expression, Chromatographic purification, ELISA

## Abstract

**Background:**

According to recent studies, the phospholipase A2 receptor 1 (PLA_2_R1) may be used as a biomarker to diagnose idiopathic membranous nephropathy (iMN). Moreover, the immune-dominant regions of PLA_2_R1 have been identified. The aim of the present study was to construct a diagnostic antigen based on the immune-dominant region of PLA_2_R1 and develop a specific serological detection method for PLA_2_R1 antibodies.

**Results:**

The tandem multi-epitope diagnostic antigen (designated ‘R101’), which includes aa 39–130 (CysR), aa 238–356 (CTLD1), and aa 1136–1234 (CTLD7) of PLA_2_R1; thioredoxin at the N-terminus; and a His tag at the C-terminus, was prepared at a concentration of 2.36 mg/mL and purity of 97.32% using *Escherichia coli* expression and affinity and anion exchange chromatography purification. The integrity and antigenicity of the R101 protein was demonstrated by western blot analysis using anti-Trx, anti-His, and anti-PLA_2_R1 monoclonal antibodies as the primary antibodies. By analysing 120 positive serum samples identified by biopsy-proven iMN (gold standard) and 240 negative samples identified by an established ELISA based on R101 protein, we concluded that the cut-off value, *kappa* value, sensitivity, specificity, and agreement rate were 0.305, 0.881, 91.67, 96.25, and 94.72% respectively. The receiver operating characteristic (ROC) curve illustrated that the diagnostic accuracy and practicability of the ELISA was excellent. The area under the curve was 0.986.

**Conclusions:**

Using prokaryotic expression and chromatography purification, immune-dominant regions of PLA_2_R1 with excellent antigenicity can be prepared and applied to serological detection of PLA_2_R1 antibodies.

## Background

With the advent of biomarkers in clinical fields, it is expected that some will be used for surveillance of disease progress rather than for complicated clinical tests (e.g. biopsy). As an autoantigen, phospholipase A_2_ receptor 1 (PLA_2_R1), a biomarker of idiopathic membranous nephropathy (iMN), plays an important role in adult nephrotic syndrome, which has variable natural history and disease progression [1]. Previous studies demonstrated that PLA_2_R1 could stimulate the immune response as a target antigen. The level of antibodies against M-type PLA_2_R1 was significantly high for primary membranous nephropathy (pMN) and there was a correlation between anti-PLA_2_R1 serum levels and disease activity [2–4]. Beck et al. identified M-type PLA_2_R1 as the target antigen in patients with iMN [2]. Subsequently, anti-PLA_2_R1 autoantibodies were confirmed to exist in 53–80% of patients with iMN [5]. Additionally, the correlation between anti-PLA_2_R1 antibody titres with clinical outcomes was reconfirmed. Furthermore, it was confirmed that the presence of distinct epitopes of PLA_2_R1 was related to disease severity and renal prognosis [5]. Therefore, disease severity may be practically monitored by detecting anti-PLA_2_R1 antibodies in the sera of patients.

The development of detection systems is a lengthy process. Initially, Beck et al. developed an anti-PLA_2_R1 antibody detection method using non-reducing sodium dodecyl sulphate-polyacrylamide gel electrophoresis (SDS-PAGE) followed by western blot analysis [2]. Although the sensitivity of western blot analysis is above 90%, it is difficult to perform outside of the laboratory and is not suitable for large numbers of samples. Later, a recombinant cell-based indirect immunofluorescence assay (RC-IFA) was developed. Until recently, an RC-IFA based on full-length human PLA_2_R1 produced by the human cell line HEK293 was widely used [6]. This assay was successfully used for disease diagnosis and surveillance and was based on the level of anti-PLA_2_R antibody. More recently, instead of RC-IFA, an enzyme-linked immunosorbent assay (ELISA) protocol was developed that used a recombinant version of the extracellular domain of PLA_2_R1 as the substrate [7].

PLA_2_R1 is a complex membrane receptor with a 10-domain extracellular region, a cysteine-rich domain (CysR), a fibronectin type II domain (FNII), and eight distinct C-type lectin domains (CTLD1–8) [8]. Each domain is independent due to the presence of small 10-amino acid linkers. Recently, several epitopes in PLA_2_R1 that were targeted by anti-PLA_2_R1 antibodies were identified [2]. Kao et al. first found that only a complex comprising the CysR, FNII, and CTLD1 domains of PLA_2_R1 under nonreducing conditions could react with sera from patients [9]. Furthermore, Fresquet et al. [10] and Seitz-Polski et al. [5] demonstrated that CysR was the most important immune-dominant epitope in most patients. Meanwhile, Seitz-Polski et al. identified three reactive epitopes of PLA_2_R1 [CysR amino acids (aa) 26–164, CTLD1 aa 223–359, and CTLD7 aa 1102–1237] and confirmed the reactivity with soluble forms of each domain by both western blot analysis and ELISA [4]. These findings suggest that a recombinant protein containing the three epitopes could potentially be used for anti-PLA_2_R1 detection.

In this study, we used aa 39–130 (CysR), aa 238–356 (CTLD1), and aa 1136–1234 (CTLD7) of PLA_2_R1 to construct a multi-epitope diagnostic antigen for anti-PLA_2_R1 antibody detection. Based on this fusion protein, we developed a novel indirect ELISA. Additionally, the sensitivity and specificity of the in-house ELISA were evaluated using PLA_2_R1-related sera.

## Methods

### Serum derivations

In total, 120 positive serum samples were collected from biopsy-proven iMN patients (gold standard) at the Second Affiliated Hospital of Nanchang University, Nanchang, China (85 samples) and Jiangxi Maternal and Child Health Hospital, Nanchang, China (35 samples). In addition, 240 negative serum samples were collected from non-iMN patients at these two hospitals (150 samples from the former and 90 samples from the latter). All of the patients provided informed consent. All of the studies were approved by the Ethics Committee at the Second Affiliated Hospital of Nanchang University (No. 20170105012) and performed in accordance with national ethics regulations. The study participants were informed of the study purpose and of their right to have their information kept confidential. Written informed consent from adults and permission from parents of minors were obtained before interviewing and blood collection.

### Construction of a recombinant diagnostic antigen expression plasmid

**Fig. 1:**
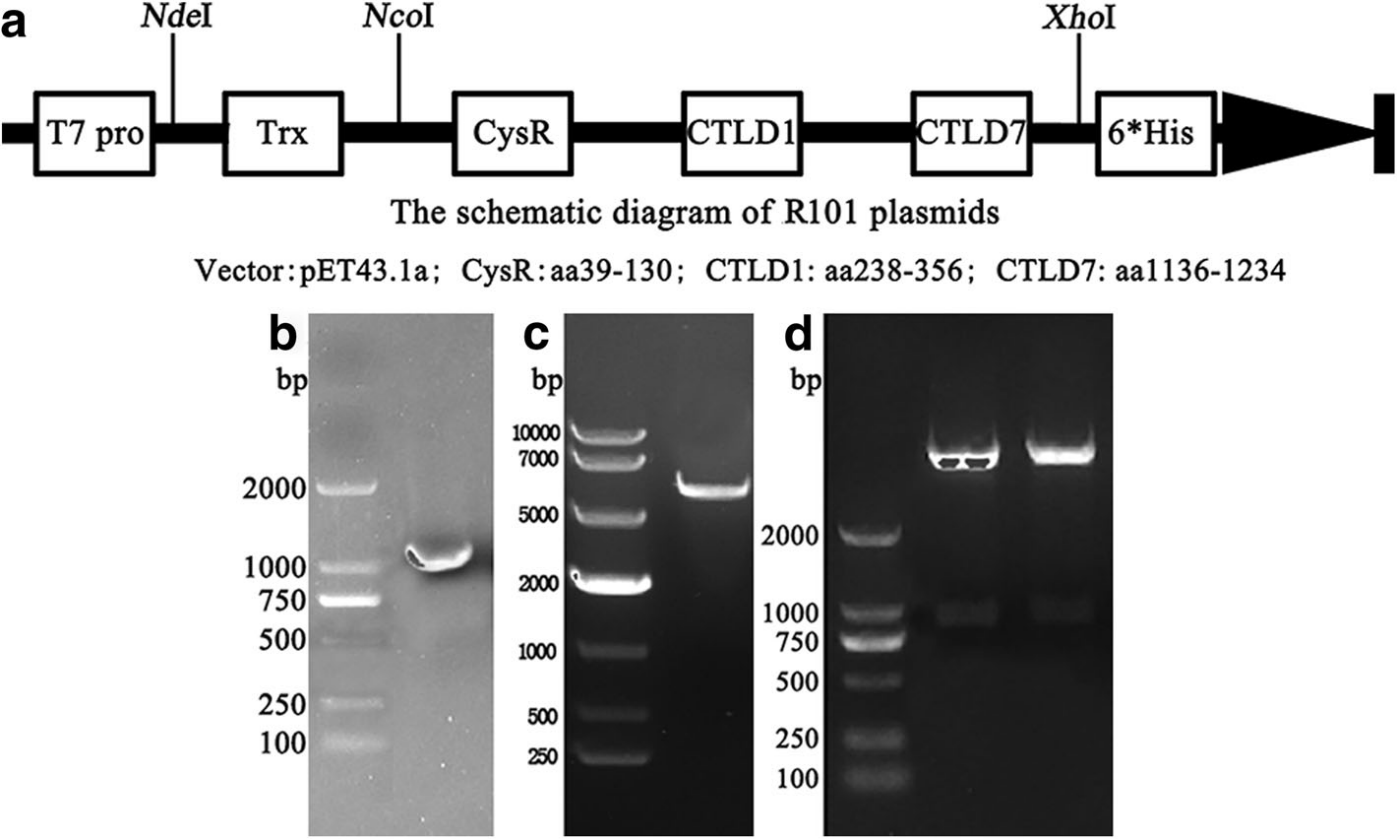
Domains of the R101 gene in the pET43.1 expression vector **a** and R101 fragment **b**. Expression vector **c** and restriction endonuclease analysis of the expression plasmid **d**, (*Nco*I and *Xho*I). Agarose gel electrophoresis showed the R101 fragment **b** and expression vector **c** obtained by dealing pMD-19 T-R101 plasmid and pET43.1a vector with *Nco*I – *Xho*I, respectively

To increase the yield of the target protein and promote the natural folding process in a prokaryotic expression system, thioredoxin (Trx) was added to the N-terminus of the fusion multi-epitope antigen. In addition, the pET43.1a vector included a His tag in the C-terminus to facilitate protein purification. Based on the findings of Seitz-Polski [5], three immune-dominant epitopes of PLA_2_R1, aa 39–130 (CysR), aa 238–356 (CTLD1), and aa 1136–1234 (CTLD7), were included in the recombinant protein. To create an expression plasmid, artificially synthesised DNA fragments encoding these three immune-dominant epitopes of PLA_2_R1 were constructed and inserted into a pET43.1a vector pre-inserted with Trx as the leading peptide (Fig. 1a). Restriction endonuclease analysis (*Nco*I and *Xho*I), small-scale expression, and sequencing were used to verify that the plasmid was constructed properly. This plasmid was designated R101 plasmid.

### Expression and purification of R101 protein

*Escherichia coli* BL21 (DE3), freshly transformed with R101 plasmid, was cultured in LB medium (10 g/L tryptone, 5 g/L yeast extract, and 10 g/L NaCl) supplemented with ampicillin (50 μg/mL) at 37 °C. When the optical density (OD) at 600 nm reached 1.0, isopropylthio-D-galactoside (IPTG) was added to a final concentration of 0.8 mM to induce R101 protein expression. The culture was incubated for an additional 4 h at 32 °C. The bacteria were harvested by centrifugation (3000×*g*, 10 min, 4 °C), re-suspended in Buffer A (10 mM Tris-HCl, 0.5% Triton X-100, pH 8.0), and then sonicated in an ice-water bath. The homogenate was centrifuged (17,400×*g*, 10 min, 4 °C) to separate the total bacterial protein, supernatant, and pellet fractions. SDS-PAGE (13.5%) was used to assess the expression level and cellular localisation of the R101 protein in *E. coli*.

After adding NaCl to a final concentration of 0.5 M, the supernatant was loaded onto an Ni^2+^ Chelating Sepharose Fast Flow chromatography column balanced with Buffer B (10 mM Tris-HCl, 500 mM NaCl, pH 8.0) for step-wise elution. The bound target protein was eluted with 300 mM imidazole and then dialysed in Buffer C (10 mM Tris-HCl, pH 8.0) for 4 h. After dialysis, DEAE chromatography was used for further purification. Finally, the eluate-containing fractions containing the highest concentration and homogeneity of R101 protein were combined and dialysed in PBS (137 mM NaCl, 2.7 mM KCl, 10 mM Na_2_HPO_4_, 2 mM KH_2_PO_4_, pH 7.4). Protein concentrations were determined using a Bicinchoninic Acid Protein Assay Kit (Sigma, MO, USA). Purified R101 protein (10 μL) was then resolved on 13.5% SDS-PAGE to assess the concentration and homogeneity of the protein.

### Western blot analysis of R101 protein

Purified R101 protein was separated by 13.5% SDS-PAGE and then transferred onto nitrocellulose membranes (Amersham, Solna, Sweden) at a constant voltage of 15 V for 20 min using a Trans-blot SD semi-dry transfer cell (Bio-Rad, CA, USA) in triplicate. After blocking non-specific antibody sites with 5% (*w*/*v*) Difco skim milk (Becton Dickinson, San Jose, MD, USA) in TBST (20 mM Tris-HCl, 500 mM NaCl, 0.05% Tween-20, pH 7.5), each of three nitrocellulose membranes were reacted with either mouse anti-Trx monoclonal antibody (1:2000; Merck Millipore), mouse anti-His monoclonal antibody (1:2000; Merck Millipore), or mouse anti-PLA_2_R monoclonal antibody (1:2000; Abcam). The secondary antibody was a goat anti-mouse IgG-HRP conjugate (1:4000; Merck Millipore). After washing with TBST three times and TBS (20 mM Tris-HCl, 500 mM NaCl, pH 7.5), 3,3′-diaminobenzidine tetrahydrochloride (DAB) substrate solution (Sigma) was added and the reaction was quenched with distilled water.

### Indirect ELISA for detecting anti-PLA_2_R1 antibodies

Based on R101 protein, a novel indirect ELISA was established to detect anti-PLA_2_R1 antibodies in sera identified to be iMN-positive by clinical and laboratory findings. Briefly, microplates were coated with 100 μL of R101 solution (5 μg/mL) diluted in coating buffer (0.05 M carbonate/bicarbonate, pH 9.6) per well and incubated overnight at 4 °C. The microplates were then blocked with blocking buffer (5% non-fat dry milk and 0.05% Tween-20 in PBS) for 1 h at 37 °C. Next, 100 μL of sera (1:10 dilution in PBS) was added to the R101-coated wells in duplicate, and incubated for 1 h at 37 °C followed by washing. Bound antibodies were detected by incubation with 50 μL of goat anti-human IgG-HRP conjugate antibody (1:2000 dilution in blocking buffer) for another 30 min at 37 °C followed by washing. Then, 50 μL of 3,3′,5,5′-tetramethylbenzidine (TMB) substrate (MP, CA, USA) was added and incubated for 15 min. Finally, the reaction was quenched and the OD values were measured at 450 nm with a microtitre plate spectrophotometer, with a reference wavelength of 620 nm. The cutoff value was defined as 2.1 times the average OD value of negative sera. Sera with an OD value ≥ the cutoff value were defined as positive, while sera with an OD < the cutoff value were defined as negative.

## Results

### R101 protein with high antigenicity was prepared by prokaryotic expression and chromatography purification

**Fig. 2:**
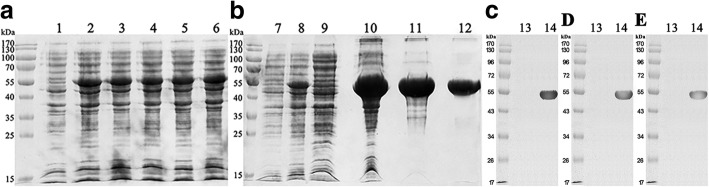
SDS-PAGE analysis of small-scale expression **a**, large-scale expression and purification steps of R101 protein **b**, and western blot analysis of R101 protein* **c**, **d**, **e**. Lane 1, non-induced bacteria; lanes 2–6, expression levels of different single colonies; lane 7, non-induced bacteria; lane 8, total bacterial proteins after sonication; lane 9, flow-through fraction from affinity chromatography; lane 10, supernatant from the homogenate; lane 11, eluates washed with 300 mM imidazole in affinity chromatography; lane 12, eluates washed with 200 mM NaCl in DEAE chromatography; lane 13, non-induced bacteria; and lane 14, purified R101 protein *C. Mouse anti-Trx monoclonal antibody as primary antibody; *D. Mouse anti-His monoclonal antibody as primary antibody; and *E. Mouse anti-PLA_2_R monoclonal antibody as primary antibody. All of the secondary antibodies were goat anti-mouse IgG-HRP conjugates.

The artificially synthesised R101 fragment obtained by double-restriction endonuclease treatment (Fig. 1b, *Nco*I and *Xho*I) was inserted into a pET43.1a vector with a pre-inserted Trx tag with the same restriction sites (Fig. 1c). The expression plasmid was identified by restriction endonuclease analysis (Fig. 1d, *Nco*I and *Xho*I) and sequencing. Moreover, small-scale expression of R101 protein in *E. coli* showed that the molecular weight of the target protein was ~ 52.6 kDa (Fig. 2a, lanes 2–6) compared with non-induced bacteria (Fig. 2a, lane 1) as expected. Together, these results suggest that the plasmid was constructed properly with expected antigen expression in the *E. coli* DE3 strain.

The percentage of R101 protein in the total bacterial protein reached 22.41% (Fig. 2b, lane 8) in large-scale fermentation and SDS-PAGE analysis showed that the protein existed mainly in the soluble form (Fig. 2b, lane 10). Additionally, R101 protein bound easily to the column matrix during affinity chromatography and was eluted with 300 mM imidazole in Buffer C. The concentration and homogeneity of R101 were 3.18 mg/mL and 93.64%, respectively (Fig. 2b, lane 11). After dialysis in Buffer A, the protein was loaded onto a DEAE chromatography column. Finally, it was eluted with 200 mM NaCl in Buffer A. The final concentration and homogeneity of R101 protein were 2.36 mg/mL and 97.32%, respectively (Fig. 2b, lane 12).

With anti-Trx (Fig. 2c), anti-His tag (Fig. 2d), and anti-PLA_2_R monoclonal antibodies (Fig. 2e) as the capture antibodies, a specific signal band at ~ 52.6 kDa was detected on the nitrocellulose membrane as expected, consistent with the molecular weight of the R101 protein containing the three related domains. In addition, western blot analysis demonstrated that the R101 protein was the diagnostic antigen.

### Novel anti-PLA_2_R1 antibody ELISA based on R101 protein distinguishes sera from iMN patients and non-iMN patients

**Fig. 3:**
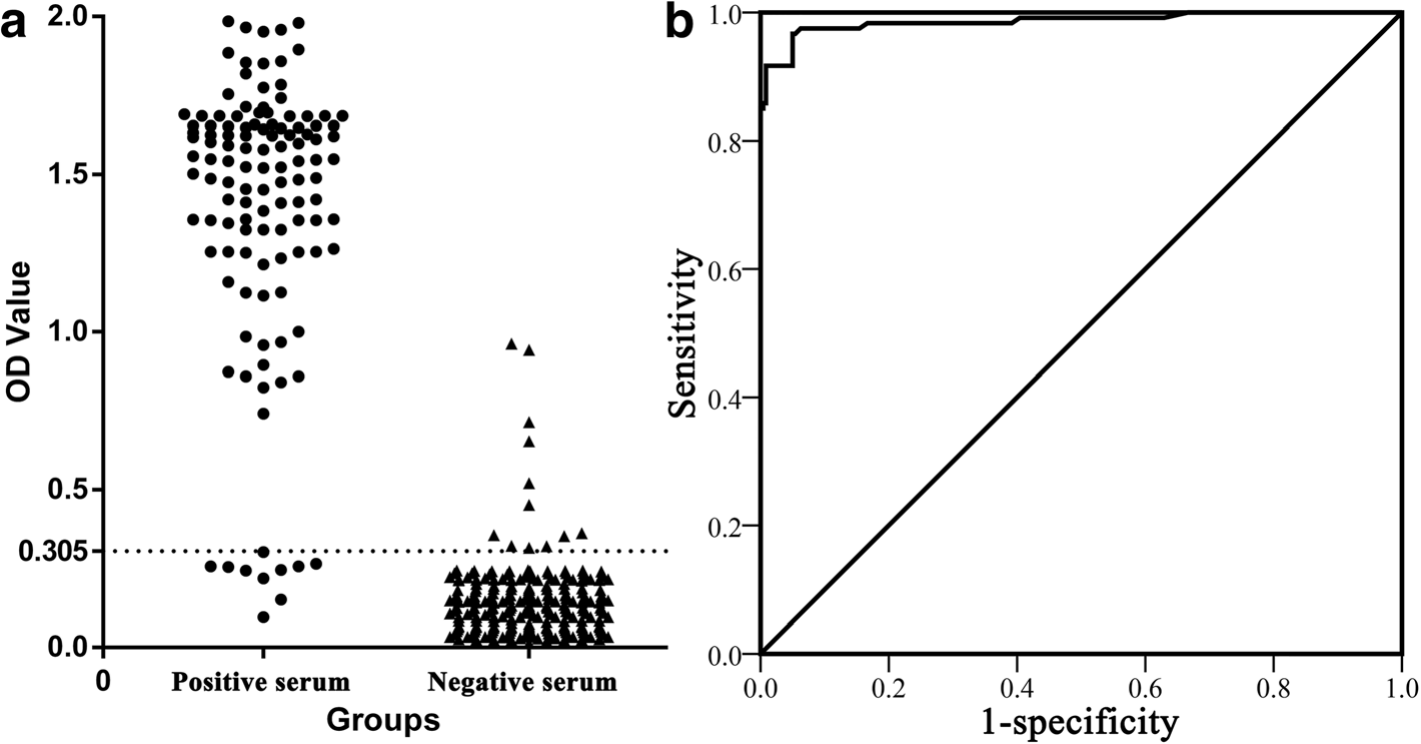
Scattergram **a** and ROC curve **b** from ELISA detection

Based on R101 protein, we established a novel indirect ELISA and evaluated it using PLA_2_R1-related sera. We used the ELISA to analyse a total of 360 serum samples (120 positive and 240 negative sera). The OD values in the positive and negative serum groups were 1.387 (1.307–1.467; 95% confidence interval) and 0.145 (0.130–0.160), respectively and could be distinguished from each other (*p* < 0.01) (Fig. 3a). According to that of 2.1 times the average OD value of negative sera, the cutoff value for the novel ELISA was 0.305. According to the data, the agreement rate (π) = 94.72%, *χ2* = 0.00, *p* > 0.05 and *kappa* = 0.881. This suggests that a statistically significant difference did not exist between the novel ELISA and biopsy results and they were perfectly consistent. The receiver operating characteristic (ROC) curve showed that the diagnostic accuracy and practicability of the ELISA was excellent (Fig. 3b). The area under the curve was 0.986, with a sensitivity of 91.67% and specificity of 96.25%.

## Discussion

Previous studies revealed the diagnostic and biomarker value of anti-PLA_2_R1 antibodies in iMN disease surveillance [5, 10]. Moreover, the immune-dominant epitopes were also identified [5]. However, owing to lack of a diagnostic antigen, serological tests such as ELISA for detecting anti-PLA_2_R1 antibodies are still in development. In this study, we expressed and purified an epitope-based diagnostic antigen based on the immune-dominant epitopes CysR, CTLD1, and CTLD7 of PLA_2_R1. The antigenicity of these related epitopes has been previously demonstrated. We hoped that the fusion protein would react with the sera of iMN patients. Interestingly, several common genetic variants associated with iMN have been localised to these domains [11].

In theory, intact PLA_2_R1 is difficult to express in *E. coli* since PLA_2_R1 is cysteine rich, especially in the CysR domain, which is the immune-dominant epitope [8]. There are 54 cysteines in the extracellular domain of PLA_2_R1. After determining the location of the immune-dominant epitopes, we retained only five pairs of cysteines responsible for the conformation to reduce the mismatch of disulfide bonds. By incorporating Trx, determination of epitopes, and optimisation of the expression conditions, we obtained a high yield of soluble R101 protein, which ensured the formation of the native conformation of the protein. Our results also demonstrated that R101 protein could be produced in a soluble form on a large scale (Fig. 3). In addition, a His tag in the C-terminus of the fusion protein facilitated the purification procedure. After only two steps of chromatographic purification, the target protein was obtained with concentration and homogeneity values of up to 2.36 mg/mL and 97.32%, respectively.

The reactivity of R101 with positive serum is vital in an ELISA. Fresquet et al. [12] recently demonstrated that the CysR domain alone contains an anti-PLA_2_R1 epitope, while Kao et al. [9] suggested that one or several epitopes are intertwined between the CysR and CTLD1 domains. Seitz-Polski et al. [5] confirmed the presence of three independent epitopes by demonstrating the presence of three autoantibodies that recognised CysR, CTLD1, and CTLD7. Herein, we selected aa 39–190 of CysR, aa 238–356 of CTLD1, and aa 1136–1234 of CTLD7 for our study. Western blot (Fig. 2c-e) and ELISA results confirmed the high antigenicity of the R101 protein by demonstrating it could bind to anti-PLA_2_R1 antibodies.

## Conclusions

We successfully used prokaryotic expression and chromatographic purification to purify R101 protein, which includes the three immune-dominant regions of PLA_2_R1. Furthermore, R101 protein was able to detect anti-PLA_2_R1 antibody. This protein may be used to establish a robust ELISA for the detection of anti-PLA_2_R1 antibodies in patients with iMN, and creates a link between a serological biomarker and the disease.

## Abbreviations

CTLD: C-type lectin domains
CysR: cysteine-rich domain
FNII: fibronectin type II domain
iMN: idiopathic membranous nephropathy
IPTG: isopropylthio-D-galactoside
PLA_2_R1: phospholipase A_2_ receptor 1
pMN: primary membranous nephropathy
RC-IFA: recombinant cell-based indirect immunofluorescence assay
